# Book Reviews

**DOI:** 10.1155/S1463924679000791

**Published:** 1979


**Meeting Reports** *(Continued from page 287)*

determination of viscosity by flow injection analysis.

The final lecture session of the conference was chaired by Dr. Betteridge and started with a paper delivered by Dr. K. Brunt on the design of an amperometric flow detector based on a rotating disc electrode. This was followed by a paper from Dr. M. Trojanowicz of the University of Warsaw who described a system for the continuous determination of sulphate using a differential flow system. The final paper, by Dr. Alan Townshend from Birmingham, described an extremely novel system enabling chemiluminescent detectors to be applied to flow injection.

Dr. Betteridge then gave some light-hearted suggestions for solving the problem of nomenclature, after which Dr. van der Linden presented flowers to Ms. Ellen Abeling and Mrs. Tilly Sijpesteyn who had been responsible for running the conference office and who had provided much invaluable help.

Finally, the conference was formally closed and everyone came away having learned a great deal about the state of the art of flow analysis, and having spent a very pleasant week in one of the most beautiful cities of Europe.

Proceedings of the conference will appear in a special edition of Analytica Chemica Acta to be published shortly.

**Tim Sly**

### Book Reviews

### Laboratory Engineering and Manipulations: 3rd Edition

*Edited by E.S. Perry and A. Weinsberger. John Wiley & Sons, Chichester, 1979, pp 531 + xi, £26.80*
*ISBN 0471-032-751*

In this, the third edition, the work has been reorganised and most parts have been thoroughly revised and rewritten. The chapter on 'Operations with Gases' has been deleted as little progress has been made in this technique since the previous edition. The work is now composed of eight chapters, each contributed by an expert or group of experts and covering a fundamental aspect of laboratory engineering, and each is subdivided into subject sections.

As an example the first chapter 'Selection of Materials for the Construction of Equipment' starts with a short introduction describing mechanical, electrical and thermal properties of materials; continues with a fairly detailed account of the corrosion of metals and the ways in which protection against corrosion may be achieved; briefly looks at non-metals, and concludes with a section dealing with the practical evaluation of chemical resistance. Included are tables referring to the general chemical resistance of common metals and general properties of a number of non-metals.

Other chapters are titled 'Laboratory Heat Transfer', which covers the whole field of temperature measurement and control, heating, cooling and insulation; 'Grinding, Blending, Screening and Classifying', 'Pumps and Flow Measurement'; 'Glove Box Technique'; 'Mixing'; 'Vacuum Technique'; and 'Solvent Removal, Evaporation, and Drying'. In each of these major aspect chapters a mathematical description is given where it appears necessary, types of equipment ranging from simple laboratory scale to small industrial scale are described and illustrated, and an extensive list of references on 'Solvent Removal' deals with the subject in a too mathematical way with little information on available equipment. The index of twenty-three pages would appear to be perfectly adequate.

The book is well produced with numerous figures, tables and illustrations of a consistent quality. It is doubtful whether it would find much use in the small analytical laboratory except as background reading, but in a larger organisation its depth of detail on such a wide range of subjects would make it a well used reference book.

*T.G. Alliston*

### Product News

### Desk-top computer

Hewlett-Packard have introduced the latest addition to their range of modular desk-top computers, the System 45B. System 45B features a standard memory of 56266 bytes, which is expandable up to a maximum of 448,906 bytes. The operating system is held in its own room so all this memory is available to the user. It also has built-in mass storage with two tape cartridge drives (one optional).

Other features include a 31 cm CRT with a standard alphanumeric mode and a graphics option. In the alphanumeric mode, the CRT can display up to 1920 characters, and the graphics option can be used to generate and display data plots, two and three dimentional drawings, charts, etc. A copy of the display can be made by the internal printer/plotter. A range of standard peripherals can also be linked to the system. System 45B has a typewriter-style keyboard with alphanumeric control, editing keys, and 32 user-definable, special function keys, and uses enhanced ANSI BASIC language. In addition to the standard keyboard, optional keyboards with special characters for German, French, Spanish and Scandanavian languages are available. The Hewlett-Packard library of software packs enables the system to be quickly tailored to application requirements such as engineering design, business management, statistical analysis, financial planning and instrument control.

*Hewlett-Packard Ltd., King Street Lane, Winnersh, Wokingham, Berks RG11 5AR, UK.*

### Karl Fischer titrator

The Photovolt Aquatest Mk IV Automatic Karl Fischer Titrator is available in the UK through Auriema. It provides a direct digital readout of the water level in liquids and solids within the range 10μg to 10mg. A microprocessor control provides a simple moisture determination. Water is titrated by electrolytic generation of Karl Fischer reagent. The inclusion of microprocessor facilities in the instrument eliminates the need for standardisation, the need to add or measure reagents and correction due to interfacing reactions. It also eliminates lengthy calculations.

Other features included are the facility to provide a programmable extraction time, automatic correction for interfering reactions, and automatic blank correction when extracting. Analyses can be carried out in the presence of anti-oxidants and compensation is made for small reactions due to carbonyl compounds.

*Auriema Ltd., 442 Bath Road, Slough SL1 6BB.*

### $CO_2$ analyser

Vickers Medical have developed a $CO_2$ analyser for use in operating theatres and intensive care units. The CD-300 analyser measures end-tidal $CO_2$ concen-

